# Bosutinib Therapy in Patients With Chronic Myeloid Leukemia: Practical Considerations for Management of Side Effects

**DOI:** 10.6004/jadpro.2016.7.2.3

**Published:** 2016-03-01

**Authors:** Patricia S. Ault, John Rose, PharmD, Lisa A. Nodzon, PhD, Elizabeth S. Kaled

**Affiliations:** 1 MD Anderson Cancer Center, University of Texas, Houston, Texas;; 2 Pfizer Inc, Collegeville, Pennsylvania;; 3 Moffitt Cancer Center, Tampa, Florida

## Abstract

The past decade has witnessed great advances in the treatment of chronic myeloid leukemia (CML), brought about in large part by the development of BCR-ABL tyrosine kinase inhibitors (TKIs). Bosutinib joins the armamentarium of approved TKIs for the treatment of chronic phase (CP), accelerated phase (AP), and blast phase (BP) Philadelphia chromosome (Ph)–positive CML resistant to or intolerant of prior therapy. Bosutinib has an adverse-event (AE) profile distinct from that of other TKIs. Diarrhea is the predominant toxicity associated with bosutinib treatment; other commonly reported nonhematologic AEs include rash and liver enzyme elevations. Cardiac events, fluid retention, and electrolyte abnormalities are infrequent. Optimal response to bosutinib requires adherence, which depends, in part, upon optimal management of associated toxicities. The oncology clinician can facilitate this process by providing patient education, timely patient follow-up, and close monitoring to promptly identify and manage AEs. Thus, optimal patient management requires a thorough and current understanding of toxicity profiles and AE management paradigms. This review provides an overview of bosutinib safety data derived from ongoing clinical trials and offers practical clinical strategies currently used to manage toxicities associated with bosutinib treatment in patients with Ph-positive CP, AP, and BP CML.

Chronic myeloid leukemia (CML) is caused by a chromosomal translocation between the Abelson (Abl) gene on chromosome 9 and the breakpoint cluster region (BCR) on chromosome 22, resulting in the constitutively active BCR-ABL tyrosine kinase that promotes myeloid proliferation (Jain, Kantarjian, & Cortes, 2013). Whereas patients with CML were historically faced with a dismal prognosis, the BCR-ABL tyrosine kinase inhibitor (TKI) era, heralded by imatinib, has vastly decreased the numbers of patients progressing from chronic (CP) to accelerated phase (AP) or blast phase (BP) CML and has improved patient survival (Agrawal, Garg, Cortes, & Quintás-Cardama, 2010).

Despite its demonstrated efficacy, approximately 30% to 40% require additional treatment beyond imatinib therapy (O’Brien et al., 2003; Santos, Kantarjian, Quintás-Cardama, & Cortes, 2011). However, the success with imatinib provided a platform for the development of the second-generation TKIs—dasatinib (Sprycel), nilotinib (Tasigna), and bosutinib (Bosulif)—and the third-generation TKI ponatinib (Iclusig), which collectively offer the potential for improving outcomes even further (Cortes et al., 2011, 2012b, 2012c, 2013a; Giles et al., 2013; Ibrahim et al., 2011a; Kantarjian et al., 2012; Khoury et al., 2012; Larson et al., 2012; Santos et al., 2011; Shah et al., 2010). 

The second- and third-generation TKIs offer patients the potential for durable cytogenetic response measured in terms of years as well as clinically meaningful improvements in health-related quality of life (HRQOL; Efficace et al., 2012; Milojkovic et al., 2012; Trask et al., 2012). Both dasatinib and nilotinib are approved for first-line treatment of patients with Philadelphia chromosome–positive (Ph+) CP-CML and for second-line disease and beyond in patients with Ph+ leukemia with resistance to or intolerance of prior therapy (Bristol-Myers Squibb, 2015; Novartis, 2015b). Ponatinib is indicated for the treatment of patients with CML or Ph+ acute lymphoblastic leukemia (ALL) who have the T315I mutation or for whom no other TKI treatment is indicated (ARIAD Pharmaceuticals, 2015).

Despite the efficacy reported with dasatinib, nilotinib, and ponatinib, each of these TKIs is associated with potentially serious complications that may preclude their use in certain patient populations (ARIAD Pharmaceuticals, 2015; Montani et al., 2012; Quintás-Cardama et al., 2009; Sano et al., 2012; Bristol-Myers Squibb, 2015; Novartis, 2015b).

Bosutinib is an orally active dual Src/Abl TKI that was approved in 2012 in the United States for the treatment of patients with Ph+ CML resistant to or intolerant of prior therapy (Pfizer Labs, 2015). Bosutinib has demonstrated activity as first-line therapy in patients with CP-CML and clinical benefit as second-line therapy in patients with CP-CML resistant to or intolerant of imatinib and as third-/fourth-line therapy in patients with CP or advanced (AP or BP) leukemia after failure of imatinib and nilotinib and/or dasatinib therapy (Brümmendorf et al., 2015; Cortes et al., 2011; Cortes et al., 2012c; Gambacorti-Passerini et al., 2010, 2014a; Khoury et al., 2012). Bosutinib has demonstrated manageable toxicities in each of these treatment settings, with the most common toxicity being diarrhea (Table 1; Pfizer Labs, 2015; Gambacorti-Passerini et al., 2014b; Kantarjian et al., 2014). Although myelosuppression is commonly observed across most TKIs, bosutinib’s tolerability profile is distinct from that of other TKIs (Table 2; Novartis, 2015a; ARIAD Pharmaceuticals, 2015; Bristol-Myers Squibb, 2015; Novartis, 2015b).

**Table 1:**
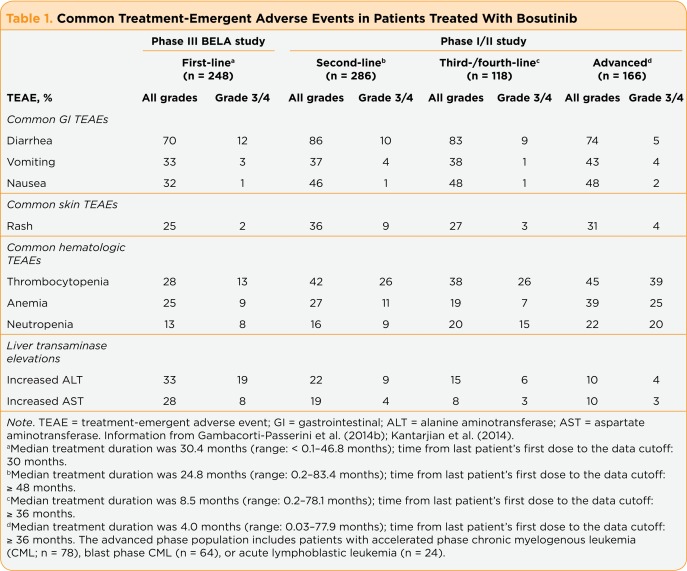
Common Treatment-Emergent Adverse Events in Patients Treated With Bosutinib

**Table 2:**
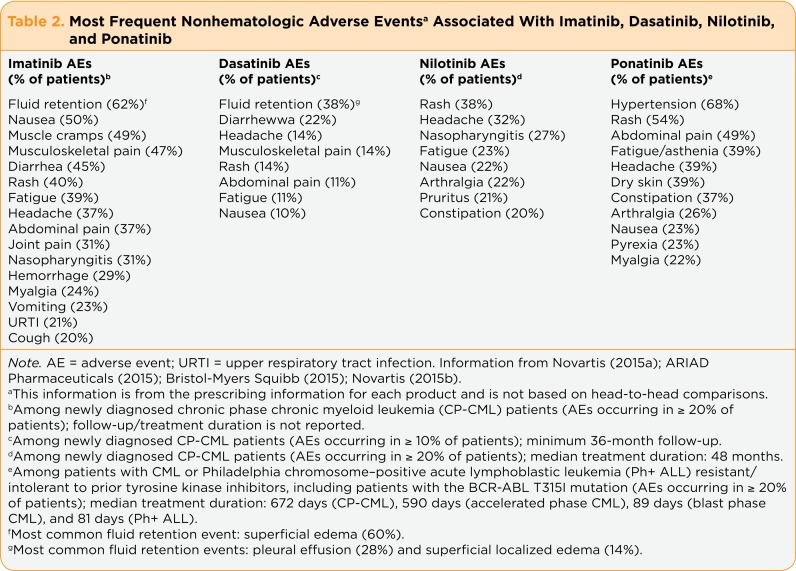
Most Frequent Nonhematologic Adverse Eventsa Associated With Imatinib, Dasatinib, Nilotinib, and Ponatinib

## CHALLENGES OF LONG-TERM USE OF TKIS

The growing number of approved and investigational TKIs available for treating CML has introduced new challenges for clinicians in deciding which agent to use as first-line therapy and as second-line/subsequent therapy (Table 3; Marin, 2012). Issues of long-term TKI treatment also represent a new frontier for CML, with treatment optimization being dependent, in part, on balancing long-term efficacy, tolerability, HRQOL, and economic considerations (Cortes, Goldman, & Hughes, 2012a). It has become increasingly apparent that close, long-term monitoring of not only treatment response but also toxicity and treatment adherence are critical components of the routine management of TKI-treated CML (Marin, 2012; Marin et al., 2010; Wong & Mirshahidi, 2011). Effective monitoring of treatment-emergent adverse events (TEAEs) is particularly relevant to patients treated with oral TKIs, as these patients are commonly responsible for self-administering daily treatment despite the potential for associated overt toxicities.

**Table 3:**
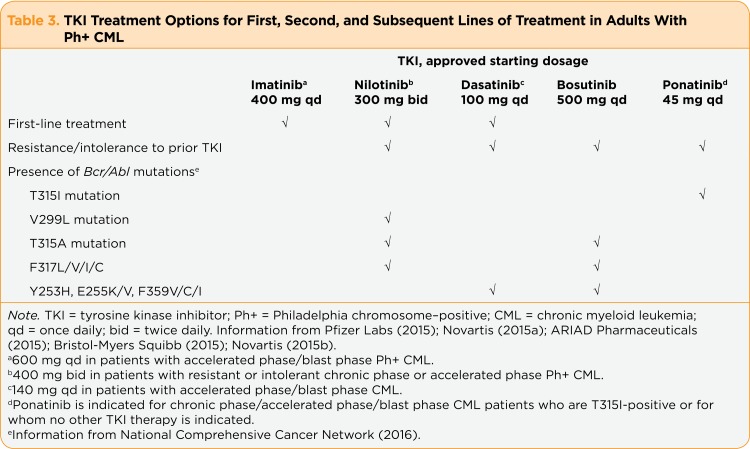
TKI Treatment Options for First, Second, and Subsequent Lines of Treatment in Adults With Ph+ CML

In a study of patients with CML receiving imatinib, adherence rates were significantly lower among patients who experienced adverse events of asthenia, muscle cramps, nausea, and joint or bone pain (Marin et al., 2010). Importantly, poor adherence to TKI treatment has been strongly linked to poor outcomes in patients with CML (Ibrahim et al., 2011b; Marin et al., 2010; Noens et al., 2009).

An understanding of the divergent toxicity profiles associated with each of the currently approved TKIs, along with knowledge of patients’ comorbidities and BCR-ABL mutational status, is critical to tailoring a particular TKI treatment to an individual patient (Cornelison, Jabbour, & Welch, 2012; Ferdinand, Mitchell, Batson, & Tumur, 2012; Wong & Mirshahidi, 2011). Although the mechanistic basis for many TKI-associated adverse events remains to be fully resolved, differences in toxicity profiles might reflect divergent specificities of the TKIs for BCR-ABL compared with other physiologically important kinases, including platelet-derived growth factor receptor (PDGF-R) and/or the c-kit proto-oncogene (Irvine & Williams, 2013; Jain et al., 2013; Puttini et al., 2006; Remsing Rix et al., 2009). Notably, bosutinib has demonstrated a lower frequency of certain toxicities, including fluid retention and bleeding disorders, relative to other second-generation TKIs.

These differences in toxicity profiles across TKIs demand caution in extrapolating from experience with managing toxicities associated with prior TKIs to guide patient management and monitoring approaches when using a different TKI. Given that oncology advanced practitioners interact closely with patients, they are well positioned to provide patient education to assist in prompt identification and management of TKI-associated adverse events.

Therefore, it is imperative that oncology advanced practitioners have a thorough and current understanding of the differences in toxicity profiles across TKIs and adverse event management strategies to facilitate treatment adherence. This review provides an overview of the practical clinical strategies currently used to manage toxicities associated with bosutinib treatment in patients with Ph+ CP-, AP-, or BP-CML.

## PRINCIPLES TO GUIDE MANAGEMENT OF CML IN THE TKI ERA

The rapid and ongoing advancements in the treatment of CML require continual update of clinical practice guidelines, including those from the National Comprehensive Cancer Network (NCCN) and the European LeukemiaNet (ELN; Baccarani et al., 2013; NCCN, 2016). There are a number of established and proposed recommendations for defining treatment response in patients with CML.

The current practice standard is attainment of complete cytogenetic response (CCyR), which is considered the main objective of TKI therapy because of the demonstrated association between CCyR and prolonged survival (Bonifazi et al., 2001; de Lavallade et al., 2008; Druker et al., 2006; Ibrahim et al., 2011a; Jabbour et al., 2011; Marin, 2012). Achieving a sustained deep molecular response (≥ 4-log reduction in BCR-ABL1 transcript levels) to TKI treatment is also an important objective given the association with treatment-free remission and the potential for obtaining prolonged clinical outcomes (Mahon & Etienne, 2014). However, achievement of molecular response targets, including BCR-ABL1 transcript levels below the limit of detection, does not necessarily correspond to complete CML remission; thus, CCyR remains the key response outcome in clinical trial and clinical practice settings.

Along with response monitoring, effective monitoring of treatment adherence is of paramount importance in routine clinical practice (Cortes et al., 2012a; Marin, 2012). It can be argued that the assessment of treatment adherence is inherently less straightforward than assessment of disease response, and there is a paucity of guidance on approaches to prevent and detect nonadherence. Adherence issues may be identified through discussion of side-effect profiles, which then prompts discussion of any missed doses due to side effects.

Molecular monitoring is also important, as increasing BCR-ABL1 transcript levels may be an indication of nonadherence; thus, results of molecular monitoring are useful for guiding discussions surrounding treatment adherence. In addition, incorporation of patient-reported outcome (PRO) measures, which assess symptom burden from the patient’s perspective (e.g., MD Anderson Symptom Inventory [MDASI] instrument), might result in more effective monitoring (Basch et al., 2006; Williams et al., 2013).

## CHARACTERIZING AND MANAGING ADVERSE EVENTS ASSOCIATED WITH BOSUTINIB

The safety and efficacy of bosutinib were first reported in a phase I/II trial of patients with CP- CML receiving bosutinib as second-line therapy after imatinib failure, with a median treatment duration of 14.9 months (< 2-year report) and as third/fourth-line therapy after imatinib and dasatinib and/or nilotinib failure, with a median treatment duration of 8.3 months (1-year report; Cortes et al., 2011; Khoury et al., 2012).

An updated analysis of safety data from this phase I/II trial was reported in CP-CML patients receiving bosutinib as second-line therapy (n = 286; 4-year update), CP-CML (n = 118) patients receiving bosutinib as third-line therapy and beyond (3-year update), and advanced leukemia (AP, BP, and ALL; n = 166) patients resistant/intolerant to prior imatinib or to multiple prior TKIs (3-year update; Kantarjian et al., 2014). In the updated analysis, toxicities associated with bosutinib treatment in all cohorts were of mild to moderate severity, with gastrointestinal events (particularly diarrhea), skin rash, hematologic adverse events, and liver transaminase elevations being the most commonly reported TEAEs across treatment lines (Table 1; Kantarjian et al., 2014).

An update of the phase III Bosutinib Efficacy and Safety in Newly Diagnosed Chronic Myeloid Leukemia (BELA) trial compared the safety of bosutinib (n = 248) and imatinib (n = 251) as first-line therapy in CP-CML patients after > 30 months of follow-up (Gambacorti-Passerini et al., 2014b). In the phase III BELA study, the safety profile of bosutinib was distinct from that of imatinib and generally similar to that observed with bosutinib in the phase I/II trial (Table 1; Gambacorti-Passerini et al., 2014b). In both studies, bosutinib was administered at a starting dose of 500 mg/day (Cortes et al., 2011; Cortes et al., 2012c; Gambacorti-Passerini et al., 2014b; Kantarjian et al., 2014; Khoury et al., 2012). Across cohorts in the second/third/fourth-line phase I/II study of bosutinib, treatment discontinuations due to adverse events occurred at a rate similar to that reported for first-line bosutinib in the phase III BELA trial (22% vs. 25%; Gambacorti-Passerini et al., 2014b; Kantarjian et al., 2014).

In retrospective analyses of data from the phase I/II study of bosutinib, the incidence of cross-intolerance between bosutinib and prior imatinib therapy was evaluated, with cross-intolerance defined as the patient having an adverse event that led to permanent treatment discontinuation of both bosutinib and prior imatinib therapy (Cortes et al., 2011; Cortes et al., 2013b). Notably, toxicities that are commonly associated with imatinib treatment and lead to intolerance to prior imatinib therapy (eg, rash, edema) have not been experienced to the same extent by patients receiving bosutinib (Table 4; Cortes et al., 2013). These findings may alleviate concerns regarding switching from imatinib to bosutinib therapy (when appropriate) and may facilitate long-term adherence to bosutinib.

**Table 4:**
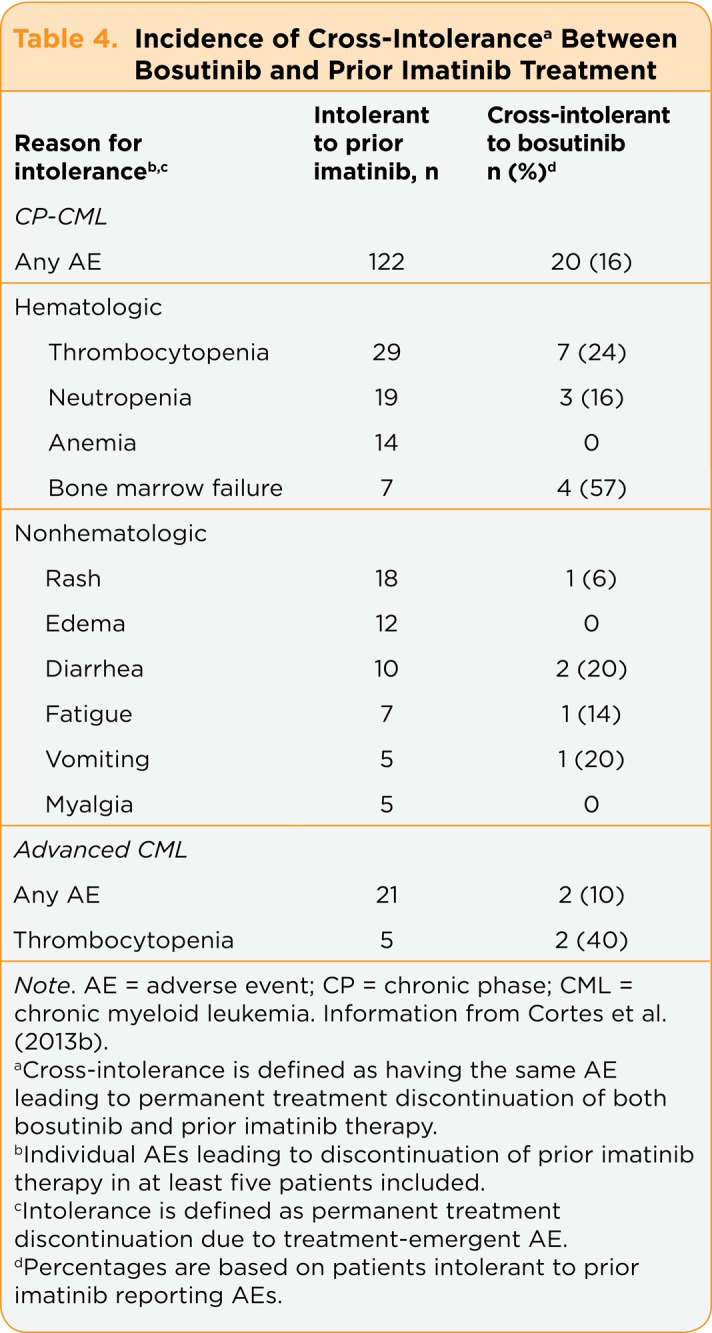
Incidence of Cross-Intolerancea Between Bosutinib and Prior Imatinib Treatment

**Diarrhea**

In the phase I/II trial of bosutinib as second/third/fourth-line therapy in patients with CML or Ph+ ALL, diarrhea was the most prominent TEAE (any grade adverse events, 82%) across cohorts irrespective of disease stage or bosutinib treatment line (Table 1; Kantarjian et al., 2014). In general, diarrhea events were of low severity (grade 3/4 adverse events, 8% of patients), first occurred early after treatment initiation (median [range] time to first event, 2.0 [1–1,330] days), and were typically limited to 2 days/any grade event (Kantarjian et al., 2014). In addition, the incidence of diarrhea was found to decrease over time on treatment (Kantarjian et al., 2014).

In keeping with the manageability of diarrhea adverse events in these patients, nearly all patients with diarrhea adverse events were maintained on bosutinib therapy; treatment discontinuation due to diarrhea occurred in only 1% of patients, despite its high incidence (Kantarjian et al., 2014). Diarrhea adverse events frequently improved spontaneously or responded to supportive measures, which mainly included concomitant medication (65% of patients), with loperamide (58%) being most commonly used (Kantarjian et al., 2014). Patients with diarrhea adverse events were less commonly managed using dose reduction and interruption (6% and 14%, respectively), thus facilitating adherence with bosutinib treatment (Kantarjian et al., 2014). Nearly all patients who underwent bosutinib dose interruption for diarrhea were successfully rechallenged with bosutinib; subsequent treatment discontinuation due to diarrhea was rare (Kantarjian et al., 2014).

Diarrhea was also the most commonly reported TEAE among newly diagnosed CP CML patients receiving bosutinib in the phase III BELA trial (Table 1), with any grade diarrhea adverse events occurring at a higher rate than in patients receiving imatinib (70% vs. 26%; Gambacorti-Passerini et al., 2014b). Whereas first events of diarrhea tended to occur early during bosutinib treatment (median time to onset, 3 days), the median time to first diarrhea event (any grade) was 53 days in the imatinib arm; the incidence of diarrhea tapered off after the first month of bosutinib treatment. Dose reductions and interruptions due to diarrhea occurred at comparable rates to those observed in patients receiving second- or third/fourth-line bosutinib in the phase I/II trial but were higher in bosutinib recipients (8%; 21%) than imatinib recipients (0%; 11%; Gambacorti-Passerini et al., 2014b; Kantarjian et al., 2014). Despite its relatively high occurrence rate, no patient discontinued treatment primarily due to diarrhea.

These findings suggest that diarrhea is generally manageable and that patients with these events can be maintained on bosutinib treatment. In the phase III BELA trial protocol, it was recommended that antidiarrheal agents such as loperamide or diphenoxylate/atropine be instituted at the first sign of diarrhea, which was found to be effective in controlling most cases of diarrhea (Cortes et al., 2012c).

Approaches for identifying and managing bosutinib-associated diarrhea, as well as patient education points, are included in Table 5; links to educational resources are listed in Table 6.

**Table 5:**
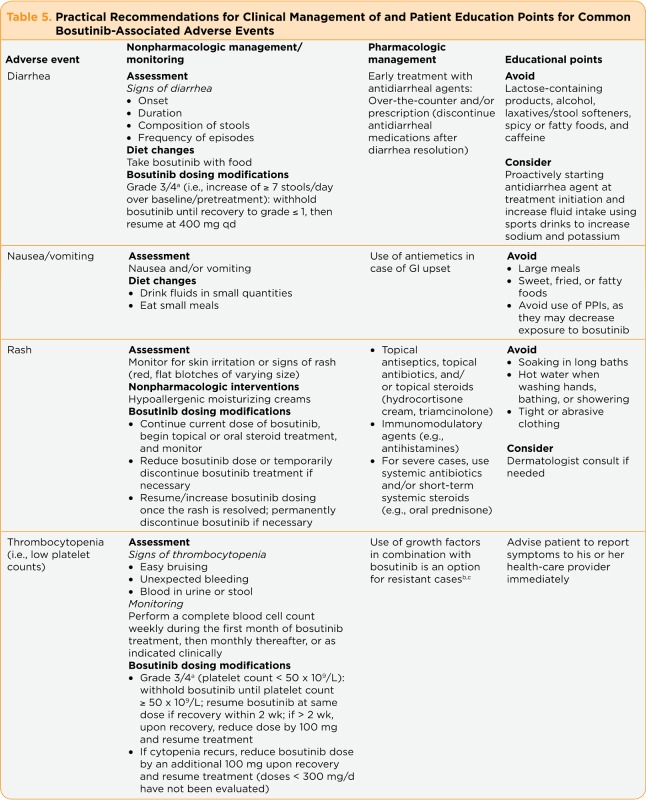
Practical Recommendations for Clinical Management of and Patient Education Points for Common Bosutinib-Associated Adverse Events

  

**Table 6:**
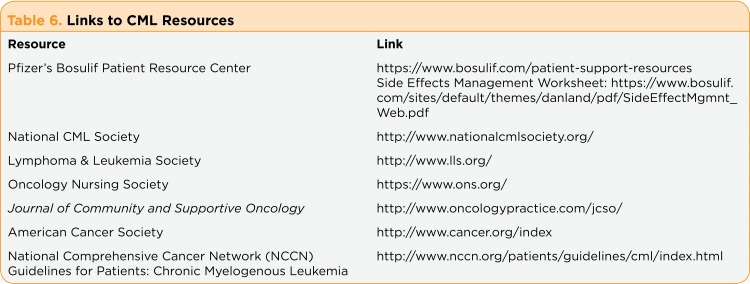
Links to CML Resources

  

**Table 5b:**
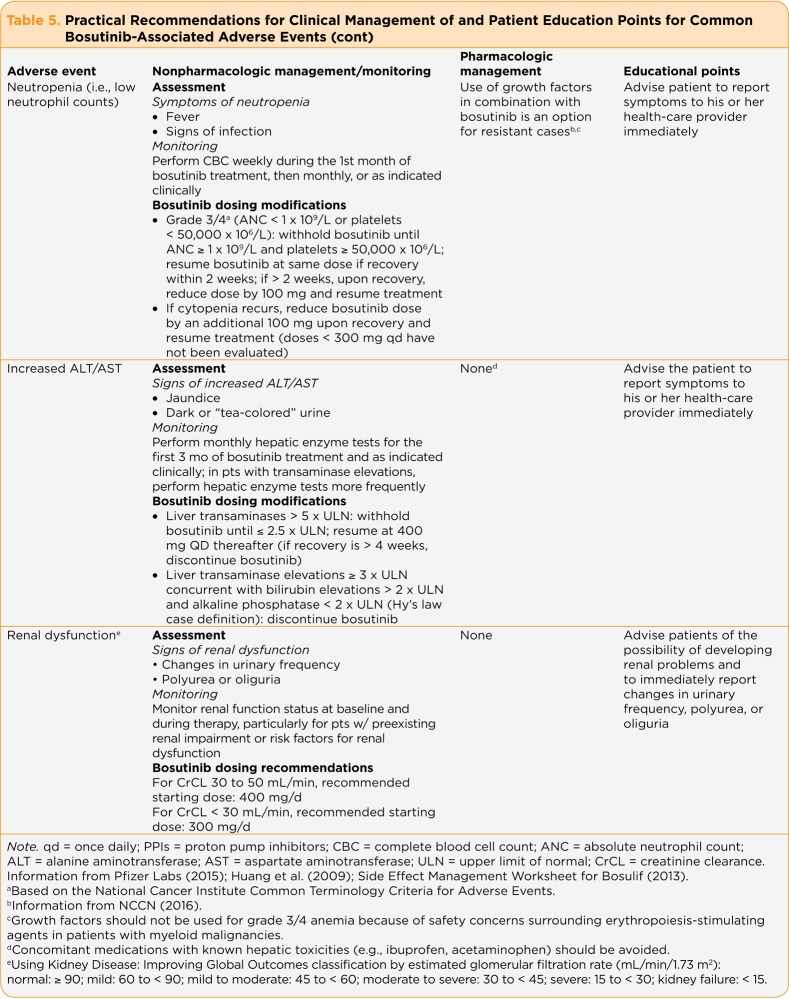
Practical Recommendations for Clinical Management of and Patient Education Points for Common Bosutinib-Associated Adverse Events (cont)

**Skin Rash**

In the phase I/II trial of bosutinib as second/third/fourth-line therapy, the incidence of rash adverse events (any grade) was 33%, including 6% at grade 3/4 severity; these adverse events occurred at similar rates across cohorts irrespective of CML phase or bosutinib treatment line (Table 1; Kantarjian et al., 2014). Among patients with CP-CML receiving second-line bosutinib after resistance/intolerance to previous imatinib, rash had a delayed onset, emerging after a median of 17.5 days, with a cumulative median duration of 25.0 days (Cortes et al., 2011). Across cohorts, only three patients (1%) discontinued bosutinib due to rash adverse events, indicating that it was generally well managed and tolerated (Kantarjian et al., 2014). In the phase III, first-line BELA trial, the incidence of all-grade and grade 3/4 rash was 25% and 2%, respectively, among bosutinib-treated patients and 20% and 1%, respectively, among imatinib-treated patients (Gambacorti-Passerini et al., 2014b).

A meta-analysis, which evaluated the incidence and clinical characteristics of rash associated with dasatinib and nilotinib, indicated that rash commonly presented as perifollicular 1–2 mm hyperkeratotic papules, which could appear on any part of the body and were often accompanied by pruritus (Drucker et al., 2013). Although the rash is typically mild, the appearance and severity of dermatologic toxicities associated with TKIs vary widely from nonerythematous papules to severe erythematous and pruritic lesions (Amitay-Laish, Stemmer, & Lacouture, 2011).

Approaches to identifying and managing bosutinib-associated skin rash are described in Table 5. General options for managing skin rash associated with TKIs include topical agents, immunomodulatory agents, systemic antibiotics for more severe cases (to prevent secondary bacterial infection), a short course of oral steroids, and interruption of the TKI followed by gradual dose escalation based on tolerability (Huang, Patel, Ahmed, Seiter, & Liu, 2009). Early treatment with oral and/or topical steroids can often alleviate symptoms.

**Hematologic Adverse Events**

Myelosuppression was frequently reported in the phase I/II trial of second/third/fourth-line bosutinib (Table 1; Kantarjian et al., 2014). Across cohorts, thrombocytopenia was the most common hematologic adverse event (any grade, 42%; grade 3/4, 30%), followed by anemia (28%; 14%) and neutropenia (19%; 14%; Kantarjian et al., 2014). Similar rates of myelosuppression were observed irrespective of CML phase or treatment line (Kantarjian et al., 2014). Median time to first myelosuppression event was 22.0 days, and the median duration/any grade event was 14.0 days, respectively (Kantarjian et al., 2014).

Patients with myelosuppression were most commonly managed by dose interruption (46%) and dose reduction (32%), but these events infrequently led to treatment discontinuation (7% of patients), emphasizing the manageability of these adverse events and the ability to maintain treatment adherence (Kantarjian et al., 2014). Among supportive interventions, growth factor support was used in 10%, and transfusions were used in 1% of patients with myelosuppression (Kantarjian et al., 2014). It should be noted that growth factors were not used in some centers participating in the phase I/II trial.

Myelosuppressive events were also common among patients receiving first-line bosutinib in the phase III BELA trial (Table 1; Gambacorti-Passerini et al., 2014b). The incidence of thrombocytopenia was similar between the bosutinib and imatinib arms (28% [grade 3/4, 13%] vs. 28% [14%]), as was the incidence of anemia (25% [9%] vs. 23% [6%]), whereas the incidence of neutropenia was lower in the bosutinib vs the imatinib arm (13% [grade 3/4, 8%] vs. 30% [16%], respectively; Gambacorti-Passerini et al., 2014b).

Approaches to identifying and managing bosutinib-associated myelosuppression are described in Table 5.

**Liver Transaminase Elevations**

Across second and third/fourth-line CP-CML and advanced leukemia cohorts in the 4-year/3-year update of the phase I/II study, alanine aminotransferase (ALT) and aspartate aminotransferase (AST) elevation TEAEs occurred in 17% and 14% of patients, respectively, with grade 3/4 ALT and AST TEAEs occurring in 7% and 3% of patients, respectively (Kantarjian et al., 2014). The median time to first ALT or AST event was 33.5 days, and the median duration of an event (any grade) was 21.0 days; the incidence of ALT events decreased over time (Kantarjian et al., 2014).

Management strategies for patients with elevated ALT and AST levels consisted of dose interruption (35%), dose reduction (18%), or concomitant medication (13%; Kantarjian et al., 2014). Among 39 patients whose dose was interrupted for ALT/AST elevations, 35 were subsequently rechallenged with bosutinib; 26 of those patients (74%) were able to continue bosutinib treatment without experiencing subsequent ALT/AST TEAEs or without treatment discontinuation due to ALT/AST TEAEs (Kantarjian et al., 2014).

Approaches to identifying and managing bosutinib-associated AST/ALT elevations are described in Table 5.

## LESS COMMON ADVERSE EVENTS WITH BOSUTINIB

**Cardiac and Vascular Adverse Events**

Although the rates of cardiac and vascular adverse events in clinical trials of patients with CML generally have been relatively low, concerns surrounding potential toxicities with CML-directed TKI therapy have arisen. In particular, an increased risk of life-threatening blood clots and severe narrowing of blood vessels was observed with ponatinib treatment, which resulted in the temporary suspension of marketing and sales of this agent in the United States, followed by a narrowing of the indication, addition of a boxed warning label, and implementation of additional safety measures to monitor patients and manage these events in 2013 (US Food and Drug Administration, 2013).

In the updated phase I/II study, cardiac TEAE rates across CP-CML second/third/fourth-line and advanced leukemia cohorts were generally low, with an all-cause cardiac TEAE incidence of 18% overall (6% considered bosutinib-related) based on the combined Medical Dictionary for Regulatory Activities (MedDRA) system organ class terms Cardiac Disorders and Investigations (cardiac and vascular terms). Most patients (10%) experienced a maximum grade 1/2 event, whereas 5% experienced a maximum grade 3, 2% a maximum grade 4, and 1% a maximum grade 5 event (Kantarjian et al., 2014). Most patients who experienced a cardiac TEAE on bosutinib treatment had prior or ongoing cardiac disorders at study entry (Kantarjian et al., 2014). The most common cardiac events were pericardial effusion (3%) and atrial fibrillation, congestive cardiac failure, tachycardia, and palpitations (2% each; Kantarjian et al., 2014). Based on electrocardiogram data, grade 3 QT interval prolongation occurred in one patient receiving bosutinib treatment, who had a grade 2 prolongation at baseline (Kantarjian et al., 2014).

Overall, patient management of cardiac adverse events included concomitant medication (40%), dose interruption (24%), and dose reduction (7%). Among the 24 patients with dose interruption, 19 (79%) were subsequently rechallenged with bosutinib, with 4 of these rechallenged patients ultimately discontinuing bosutinib treatment (Kantarjian et al., 2014). These findings indicate that most patients who experienced cardiac adverse events were maintained on bosutinib treatment. Among patients receiving first-line bosutinib in the phase III BELA trial, the incidence of cardiac TEAEs (MedDRA system organ class term: Cardiac Disorders [not including QT interval prolongation]) was similar in the bosutinib and imatinib arms (8% vs. 6%), most commonly palpitations (2% vs. 2%) and pericardial effusion (2% vs. 0; Gambacorti-Passerini et al., 2014b).

As with cardiac events, the rate of all-cause vascular TEAEs (MedDRA system organ class term: Vascular Disorders) observed in the phase I/II study was relatively low (13%); hypertension was the only vascular TEAE that occurred in ≥ 2% of patients overall (any grade, 6%; grade 3/4, 2%; Kantarjian et al., 2014). Only one patient in the third-line cohort with prior nilotinib exposure reported grade 2 peripheral arterial occlusive disease, which resolved within 10 days and was considered to be unrelated to bosutinib (Kantarjian et al., 2014). In the first-line, phase III BELA trial, the incidence of all-cause vascular TEAEs (MedDRA system organ class term: Vascular Disorders) was similar in the bosutinib and imatinib arms (10% vs. 8%), most commonly hypertension (6% vs. 4%; Gambacorti-Passerini et al., 2014b).

Although an increased risk of cardiac or vascular adverse events has not been identified with bosutinib treatment, health-care providers should be aware of the potential for cardiac and vascular adverse events associated with any TKI treatment. Thus, baseline cardiac assessments should be performed to help determine the risk status of patients and identify those in need of closer monitoring.

**Fluid Retention**

The incidence of pleural effusions with bosutinib treatment was relatively low in the phase I/II trial in patients resistant/intolerant to prior TKIs (10% [grade ≥ 3, 3%]; Kantarjian et al., 2014). Pleural effusion events were more frequent among patients in the CP-CML third/fourth-line cohort (15% [grade ≥ 3, 3%]) compared with those in the CP-CML second-line (8% [grade ≥ 3, 2%]) and advanced leukemia (10% [grade ≥ 3, 4%]) cohorts (Kantarjian et al., 2014). Median time to first pleural effusion event was 541 days, and the median duration of an any grade event was 21 days (Kantarjian et al., 2014).

Management of pleural effusions included dose reductions (26%), dose interruptions (47%), and concomitant medications (58%); four patients discontinued bosutinib due to pleural effusion (Kantarjian et al., 2014). In the update of the first-line, phase III BELA trial, pleural effusions occurred at a low incidence in both the bosutinib (4%) and imatinib (< 1%) arms, although the difference was statistically significant (p = .006, not adjusted for multiple comparisons; Gambacorti-Passerini et al., 2014b).

The current NCCN guidelines recommend the use of diuretics and supportive care for the management of fluid retention events (ie, pulmonary or peripheral edema, pleural or pericardial effusion; NCCN, 2016). It may also be beneficial to monitor closely patients with lung disease while on bosutinib treatment or other TKIs.

**Electrolyte Abnormalities**

In the updated report of the phase I/II study of second/third/fourth-line bosutinib, grade 3/4 electrolyte abnormalities with an incidence exceeding 5% were hypermagnesemia (10%) and hypophosphatemia (8%), with the former occurring more frequently in patients with prior TKI intolerance vs. resistance in both the CP-CML second-line and third/fourth-line cohorts (Kantarjian et al., 2014). Grade 3/4 electrolyte laboratory abnormalities occurring among newly diagnosed patients receiving bosutinib in the first-line, phase III BELA trial included hypophosphatemia and hypokalemia, for which the rates were lower with bosutinib (7% and 2%, respectively) vs. imatinib (22% and 6%, respectively), and hypocalcemia (bosutinib: 4%; imatinib: 2%; Gambacorti-Passerini et al., 2014b).

In patients with TKI-treated CML, it is important to capture electrolytes as part of the routine laboratory monitoring. In some cases, dietary supplements may be necessary to maintain potassium and magnesium levels, particularly given the association of these electrolytes with cardiac function.

## CASE STUDY: MANAGEMENT STRATEGIES IN ACTION

The following case study illustrates practical management strategies for the bosutinib-associated toxicities previously described.

A 50-year-old man was diagnosed with Ph+ CP-CML; front-line TKI therapy with imatinib at 400 mg daily was initiated. After 1 year, the patient had not achieved a cytogenetic response and had 80% Ph+ cells (by fluorescence in situ hybridization analysis), thrombocytosis, and neutrophilia. The patient’s disease remained minimally responsive to a standard dose of imatinib, for which the patient’s adherence to treatment was determined not to be a contributing factor. Imatinib treatment was discontinued because the patient was intolerant to dose escalation, and he was subsequently prescribed bosutinib, which was initiated at a dose of 500 mg daily.

To facilitate adherence and encourage prophylactic behavior, the patient was proactively provided with educational information on how to better manage his condition and expectations as well as possibly mitigate any potential study drug–related side effects. The patient was informed that diarrhea was the most common side effect in patients taking bosutinib, and, if it occurred, it was predominantly of low severity and transient, typically starting 1 to 2 days after initiation. The patient was advised to report any gastrointestinal events to his oncology care provider immediately, and standard-of-care interventions would be initiated at the first sign of diarrhea (e.g., concurrent antidiarrheal medication, use of sports drinks for oral hydration, and a BRAT diet [containing bananas, rice, apple sauce, and toast]).

The provider also recommended that the patient take steps to decrease the likelihood or severity of possible dermatologic side effects; they involved protecting his skin from direct sunlight, using sunscreen outdoors, avoiding alcohol-based and/or scented skin products, and keeping his skin adequately moisturized using a hypoallergenic emollient cream. At the outset, timely communication between the provider and patient was encouraged in relation to treatment adherence as well as the patient’s health and well-being.

The patient reported grade 2 diarrhea 2 days after starting bosutinib treatment. The patient also experienced grade 1 fatigue on day 5 of bosutinib treatment, which was associated with secondary dehydration caused by diarrhea. Using standard treatments, diarrhea resolved by day 28 of bosutinib treatment.

In addition, 1 week after bosutinib treatment initiation, the patient experienced grade 1 rash, which was pruritic and located on the trunk and neck. In addition to the nonpharmacologic management of his skin, the patient was treated with topical hydrocortisone cream 1% four times daily, and the rash resolved within 2 weeks.

The patient also experienced grade 1 AST elevation and grade 2 vomiting intermittently during the first year of bosutinib treatment; events of AST elevations resolved spontaneously, whereas vomiting events were treated with antiemetics; neither type of event required dose interruptions or dose reductions.

During clinic visits, adverse events and the importance of adhering to daily bosutinib therapy continued to be discussed. Within 12 months, the patient achieved a CCyR and continued receiving bosutinib therapy without recurrence of diarrhea or rash.

## CLINICAL PERSPECTIVES AND CONCLUSIONS

The oncology care provider should be aware of the toxicity profiles of available TKIs to manage such toxicities effectively. The toxicity profile of bosutinib is distinct from that of other TKIs; therefore, clinical experience gained with one TKI such as imatinib should not necessarily govern management and monitoring of adverse events in patients treated with another TKI such as bosutinib.

Diarrhea is the predominant toxicity associated with bosutinib treatment and is generally self-limiting, either with or without supportive intervention (Gambacorti-Passerini et al., 2014b; Kantarjian et al., 2014). Based on clinical experience and the results of clinical trials to date, diarrhea should be regarded as a manageable adverse event, and discontinuation of bosutinib treatment or switching to another TKI because of diarrhea is rarely necessary.

Skin rash is not unique to bosutinib, and referral to a dermatologist may be needed for comprehensive care. The other adverse events described in association with bosutinib, including hematologic and nonhematologic electrolyte abnormalities, liver enzyme elevations, fluid retention, and less frequently reported cardiac events, all have been managed in clinical trials to date.

An analysis of data from the phase I/II trial of second/third/fourth line bosutinib in patients with CML showed that the frequency of bosutinib-associated toxicities and treatment discontinuations due to adverse events among patients with CP-CML is greatest within 1 year of treatment initiation and declines over time thereafter, indicating that these patients experience an improvement in tolerability during long-term bosutinib treatment (Gambacorti-Passerini et al., 2013).

Overall, TKI-treated patients should be educated about early identification of toxicities such as diarrhea, rash, and myelosuppression to afford the opportunity for early intervention (Wong & Mirshahidi, 2011). However, educational efforts need to be supplemented with close monitoring and follow-up, particularly in the community setting, and considering the risk of nonadherence associated with oral cancer therapy. Close communication between the oncology care provider and the patient is critical for providing education on strategies for coping with bosutinib treatment–related toxicities and for setting treatment expectations. Points for patient education regarding potential toxicities associated with bosutinib treatment are provided in Table 5, and links to educational resources are listed in Table 6.

In the current TKI era, the optimal treatment of CML in patients who fail to respond to a second-generation TKI is a major outstanding question, with few guidelines and clinical trials addressing this issue to date (Jabbour, Bixby, & Akard, 2012; Keller & Brümmendorf, 2012). Optimizing strategies for the management of bosutinib-associated toxicities may facilitate adherence to bosutinib treatment and avoid unnecessary treatment discontinuations or switching between TKI treatments.

**Acknowledgments**

The authors thank Patricia A. Jordan Cole, RN, OCN®, for her clinical insights regarding the management of side effects associated with bosutinib treatment in patients with CML. Medical writing support was provided by Simon J. Slater, PhD, and Cynthia L. Gobbel, PhD, CMPP, of Complete Healthcare Communications, Inc., and was funded by Pfizer Inc.
